# An investigation into the correlation between visual performance in simulated complex environments and academic attainment among primary school students

**DOI:** 10.1038/s41598-024-56548-7

**Published:** 2024-03-11

**Authors:** Yu-Jing Tian, Chen Chen, Xiao-Han Zhang, Yu-Juan Cao, Ying-Qing Yu

**Affiliations:** 1 Department of Ophthalmology, Affiliated Children’s Hospital of Jiangnan University, Wuxi, 214000 China; 2 Department of Ophthalmology, Jiangnan University Medical Center, Wuxi, 214000 China

**Keywords:** Binocular vision function, Simulated complex environments, Rivalry-state vision test, Academic performance, Biophysics, Physiology, Psychology

## Abstract

Traditional vision screenings in schools are limited to simple visual tasks, yet students in their daily learning face more complex visual environments. Binocular rivalry tasks can partially simulate the visual challenges of real visual environments and activate advanced visual processing mechanisms that simple visual tasks cannot. Therefore, by superimposing binocular rivalry-state tasks onto simple visual tasks, we have developed an innovative vision screening program to rapidly and extensively assess students’ visual performance in complex environments. This is a cross-sectional study in which we investigated the performance of 1126 grade 1–6 students from a primary school in Wuxi, China, in rivalry-state stereoscopic vision tasks. The correlation between the screening results of 1044 students and their academic achievements was also statistically analyzed. The study results revealed pass rates of 53.5–60.5% across various visual tests. Specifically, for first-grade students, there was a statistically significant difference in standardized Chinese scores between the group that failed and the group that passed the rivalry-state stereoscopic vision test (− 0.49 ± 3.42 vs. 0.22 ± 0.58, t =  − 2.081, *P* = 0.04). This result underscores the importance of focusing on the visual adaptability of first graders in complex environments.

Trail registration: Ethics Committee of Affiliated Children’s Hospital of Jiangnan University-Certificate number: WXCH2022-04-027

## Introduction

Up to 40% of school-aged children may have vision problems leading to deficits in visual function, which are not detectable in traditional vision screening and hence are often overlooked^1–3^. When vision problems impact learning, they are referred to as “learning-related vision problems”. The American Optometric Association (AOA), in its “Clinical Practice Guidelines for the Management of Patients with Learning-Related Vision Problems”, notes that these issues can hinder an individual’s full learning potential, leading to symptoms such as poor reading efficiency, inability to maintain attention, and difficulty in switching between tasks^4^. The range of visual factors affecting learning is broad, encompassing aspects such as visual acuity^5,6^, refractive status^7,8^, ocular deviation^9^, stereoscopic acuity^9,10^, ocular motor function^11–14^, visual spatial memory^15^, and visuo-motor integration. Many domestic and international studies are limited to single visual tasks, whereas children in their daily learning encounter complex visual environments and multiple visual tasks.

In simple visual tasks, the presentation of visual information is almost linear, and in cases where binocular signals completely match, the visual system can easily merge images from both eyes by detecting corresponding luminance signals, thereby forming stereoscopic perception^16^. However, in real natural scenes, the presentation of visual information is almost always nonlinear and diversified. Such information often leads to incomplete matches between left and right eye visual signals, posing challenges to binocular fusion. For example, when observing 3D scenes, obstructions typically generate different images in the corresponding areas of each eye^17^, or intermittent deficits in visual sampling can lead to partial contour absence or even contradictions between binocular signals^18,19^. Employing slightly different binocular rivalry tasks for each eye to simulate the mismatch of binocular signals in natural environments is a viable approach. Neurophysiological studies indicate that the visual cortex’s response to simple linear visual information and rivalry-state visual information differs^20–22^. Under conditions of binocular rivalry, the brain additionally engages advanced visual processing mechanisms, using more complex algorithms to match images from both eyes, ultimately achieving binocular fusion and forming stereoscopic perception^23–26^.

**Figure 1:**
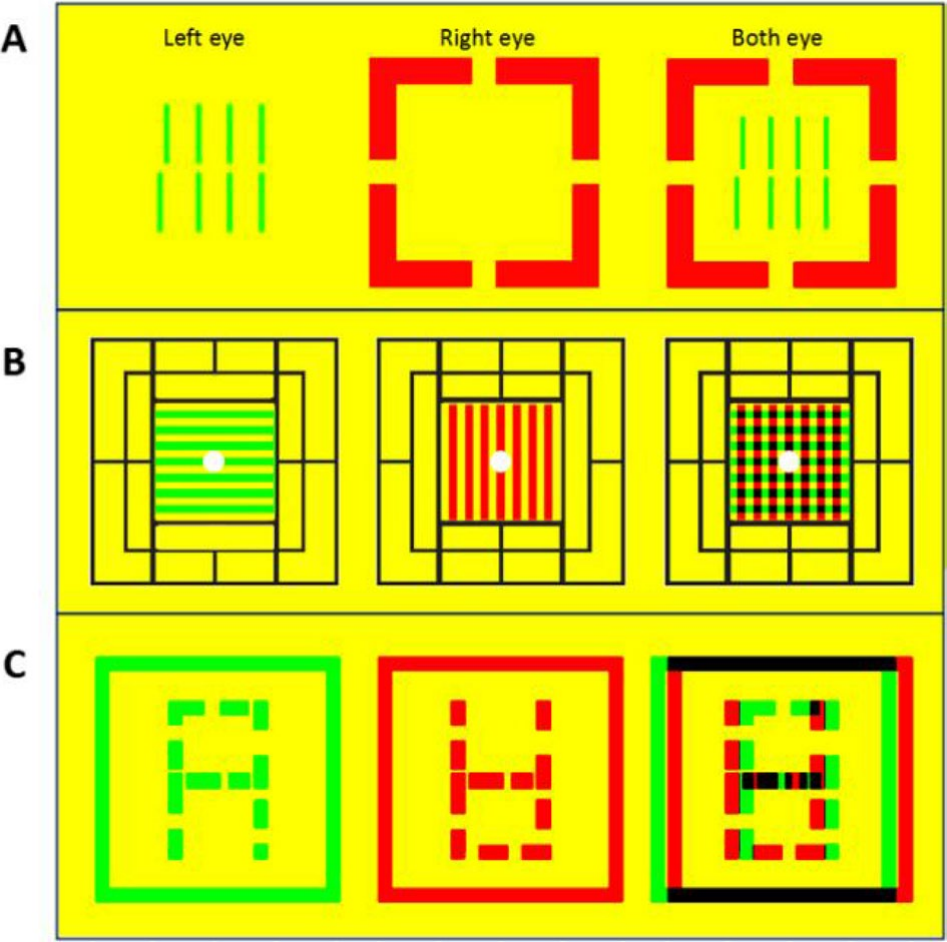
Rivalry-State Visual Task Test Methods. (**A**) Rivalry-Cursor Visual Acuity Test, where the tested eye sees two rows of parallel vertical lines in the center, and the opposite eye sees a surrounding frame. (**B**) Rivalry-Simultaneous Vision Test, where the peripheral field frame is visible to both eyes for image fusion locking, with the horizontal line visible to the left eye and the vertical line to the right eye. (**C**) Rivalry-Fusion Vision and Rivalry-Stereoscopic Vision Tests, where the images visible to the left and right eyes have complementary contours, with dynamic changes in horizontal binocular disparity.

Although the brain's response differs between simple and complex visual tasks, the simple visual tasks used in traditional school vision screenings fail to observe students' real visual performance in complex environments, which may result in overlooking actual visual problems that arise during learning. We aim to develop an innovative visual task screening program that simulates complex environments, specifically designed for large-scale and rapid assessment of students' visual performance in such environments. This program builds upon clinically common classic visual function test indicators by incorporating binocular rivalry tasks, creating a comprehensive binocular vision task mode (rivalry-state visual task). Specifically, it utilizes the cursor acuity test^27^ and a three-level binocular vision function test (simultaneous vision, fusion vision, and stereoscopic vision)^28^ as base indicators, combined with binocular rivalry-state tasks, to construct a complete visual function screening program that can assess the presence of visual distortion and risks of binocular imbalance in complex or poor visual environments. This program includes four key tests: Rivalry-cursor visual acuity test, Rivalry-simultaneous vision test, Rivalry-fusion vision test, and Rivalry-stereoscopic vision test (Fig. 1). These four rivalry-state visual task tests comprehensively evaluate students' visual adaptability to varying visual environments and determine the presence of visual problems that could hinder the full realization of individual learning potential. This study investigated the ability of primary school students from grades 1 to 6 in Wuxi, China, to handle binocular rivalry-state visual tasks, observed the overview of their visual adaptability in simulated complex visual environments, and further explored the correlation between these abilities and academic performance, in hopes of identifying early warning indicators of visual function deficits that hinder the full realization of primary students' learning potential.

## Materials and methods

### Participants

This study utilized a whole-cluster random sampling approach for a cross-sectional survey of rivalry-state visual task performance among 1126 students in a typical six-year primary school in Wuxi, China, from June to September 2022. The criteria for inclusion in the statistical analysis were: (1) The ability to complete the rivalry-state visual task tests. (2) Valid end-of-term academic grades for all subjects.

### Stimuli and procedure

#### Rivalry-state visual task test

The test was conducted in a quiet, private room with natural and constant lighting. A 32-inch adjustable-height monitor (resolution 1920 × 1080 pixels) was used to present stimulus images at a viewing distance of 80 cm. Participants wore corrective glasses (if needed) overlaid with red-blue glasses (red lens on the left, blue lens on the right) and stood upright for the test, with their eyes level with the center of the screen. (Note: This series of stimulus images can also be used on polarized 3D displays).

1. *Rivalry-Cursor Visual Acuity Test*: When testing the left eye, the right eye sees an image of a frame missing some contours (with a side angle of 4.30° at a viewing distance of 80 cm), and the left eye sees two rows of parallel vertical lines (2.51°) (Fig. 1A). In each test, one line from the two rows randomly aligns with or deviates from the position of the other parallel lines (by an offset of 0.07°). If the participant correctly judges the alignment or misalignment of the two rows of lines, they pass the test for that eye. When testing the right eye, the visible images for each eye are swapped. Passing the test with both eyes is considered a pass for this test.
2. *Rivalry-Simultaneous Vision Test*: The stimulus image consists of a surrounding frame (4.30°) and horizontal and vertical lines in the center (2.51°). The surrounding frame is visible to both eyes for fusion locking, with the horizontal line visible only to the left eye and the vertical line only to the right eye (Fig. 1B). If the participant can see both the horizontal and vertical lines simultaneously or alternately, they pass this test.
3. *Rivalry-Fusion Vision Test*: The left eye sees an image consisting of a frame (4.30°) and an ‘A’ shaped visual marker (3.22°), while the right eye sees the same frame with a complementary outline of an inverted ‘A’ visual marker (Fig. 1C). If the participant can see a number 8 formed by the fusion of the two images, they pass this test.
4. *Rivalry-Stereoscopic Vision Test*: The stimulus image, as in Fig. 1C, involves the frames in the images for the left and right eyes moving horizontally in alternating and non-alternating patterns (speed 0.14°/s, range of movement 0.36°, frequency 0.5 Hz), while the number 8 in the images moves horizontally in the opposite pattern to the frame, creating a perceptual sense of depth for the number 8 and the frame (total disparity range 0–0.72°). If the participant can judge the relative depth of the number 8 in relation to the surrounding frame, they pass this test.

### Statistical analysis

Data analysis was performed using SPSS 23.0 statistical software. The Z-score normalization method was used to process students’ Chinese and mathematics scores (using the class average as the mean). Spearman's rank correlation coefficient was used to assess the correlation between grade levels and various rivalry-state visual task test results. Based on students' results in individual visual task tests, they were divided into pass and fail groups. An independent sample T-test was used to determine the differences between groups, with *P* < 0.05 indicating statistically significant differences.

### Ethics approval

The experiments and protocols have been approved by the Ethics Committee of Wuxi Children Hospital (wxch202204027). The research complies with internationally accepted standards for research practice and reporting and has been carried out within an appropriate ethical framework.

### Consent to participate

The informed consents were obtained from the participant's legal guardians or parents.

## Results

### Group characteristics

A total of 1044 primary school students from grades 1 to 6 completed all rivalry-state visual task tests, and their final academic scores were valid. Of these, 528 were boys and 516 were girls. The overall pass rates for the Rivalry-cursor visual acuity test, Rivalry-simultaneous vision test, Rivalry-fusion vision test, and Rivalry-stereoscopic vision test across all grades were 53.5% (559/1044), 59.1% (617/1044), 60.5% (632/1044), and 54.6% (570/1044), respectively. The results of the rivalry-state visual task tests for each grade are shown in Table 1. Spearman’s rank correlation coefficient analysis indicated that there is a slight positive correlation between the Rivalry-cursor visual acuity and grade advancement (ρ = 0.114, *P* < 0.001). Similarly, the Rivalry-simultaneous vision function also showed a slight positive correlation with grade advancement (ρ = 0.108, *P* < 0.001). However, no correlation was found between grade advancement and either the Rivalry-fusion vision or Rivalry-stereoscopic vision functions.

**Table 1:** Results of rivalry-state visual task tests for primary school students in grades 1–6.

	Total N	Rivalry-cursor visual acuity test		Rivalry-simultaneous vision test		Rivalry-fusion vision test		Rivalry-stereoscopic vision test	
		N passed [%]	ρ [*P*]	N passed [%]	ρ [*P*]	N passed [%]	ρ [*P*]	N passed [%]	ρ [*P*]
Grade 1	213	108 [50.7%]	0.114 [0.000***]	115 [54%]	0.108 [0.000***]	140 [65.7%]	− 0.028 [0.371]	111 [52.1%]	0.021 [0.498]
Grade 2	214	94 [43.9%]		112 [52.3%]		120 [56.1%]		115 [53.7%]	
Grade 3	145	81 [55.9%]		90 [62.1%]		92 [63.4%]		82 [56.6%]	
Grade 4	191	92 [48.2%]		118 [61.8%]		112 [58.6%]		106 [55.5%]	
Grade 5	194	130 [67%]		113 [58.2%]		118 [60.8%]		110 [56.7%]	
Grade 6	87	54 [62.1%]		69 [79.3%]		50 [57.5%]		46 [52.9%]	

N: Number of student, *ρ*:Spearman's correlation.

***P < 0.001.

### Correlation between rivalry-state visual task test results and academic performance

**Figure 2:**
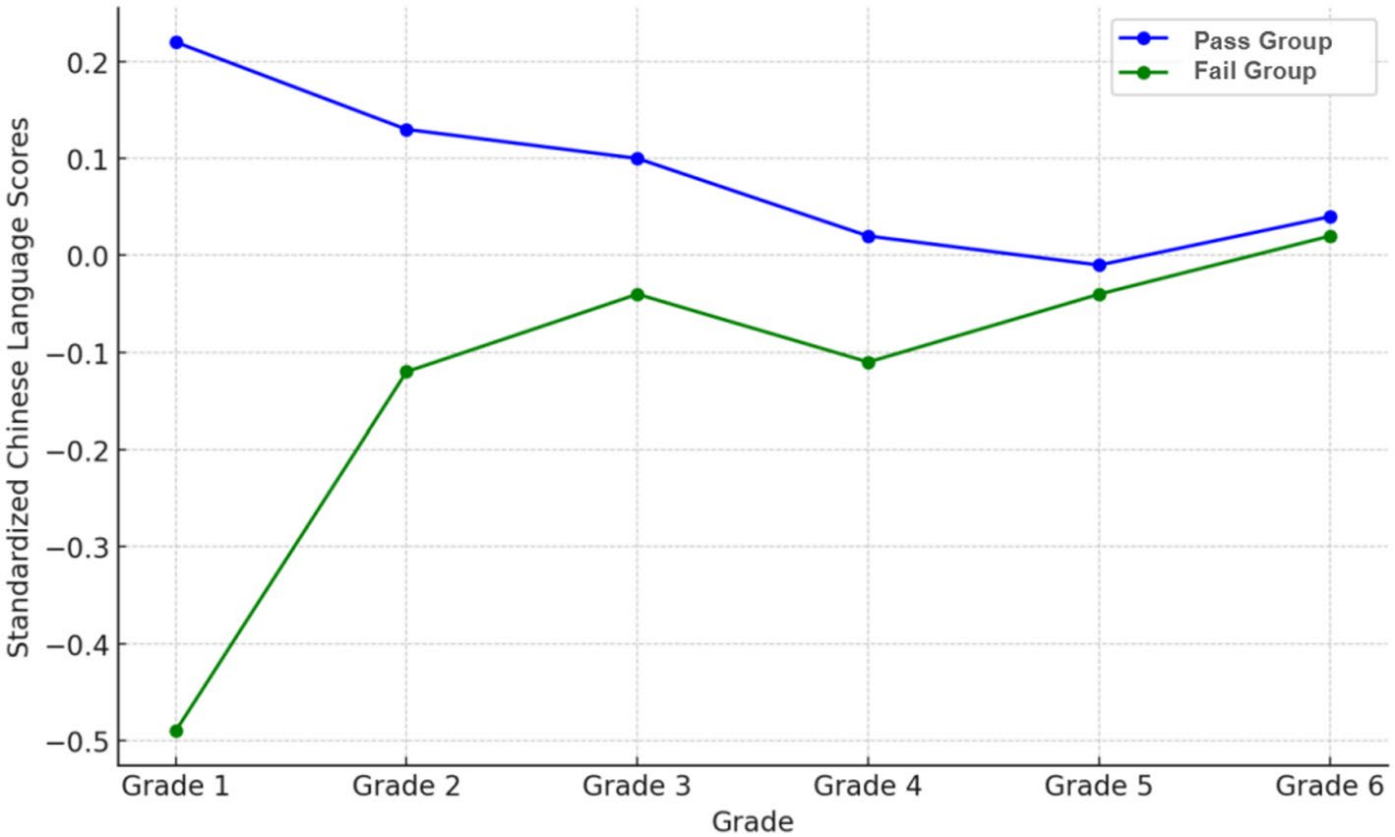
Comparison of Chinese language scores between pass and fail groups in rivalry-stereoscopic vision test across different grade levels.

Table 2 shows the correlation between various rivalry-state visual task tests and standardized Chinese language scores, as well as the comparison between the pass and fail groups. The Spearman's rank correlation coefficient between the Rivalry-stereoscopic vision test results and standardized Chinese language scores was 0.077 (*P* = 0.013), indicating a very weak positive correlation. To further analyze this correlation, we first compared the standardized Chinese language scores between the pass and fail groups in the Rivalry-stereoscopic vision test, where the fail group had an average score of -0.16 and the pass group had 0.09, showing a statistically significant difference (t = − 2.706, *P* = 0.007). We also observed this difference across different grades; overall, the fail group scored lower than the pass group in all grades, with this difference being more pronounced in the lower grades at the beginning of school, but diminishing as grades progressed (Fig. 2). Stratified analysis results (Table 3) show that the difference between the fail and pass groups was significant in the first grade (− 0.49 ± 3.42 vs. 0.22 ± 0.58, t = − 2.081, *P* = 0.04). The *P*-value of the difference between groups in the second grade was also close to 0.05 (*P* = 0.068), and this intergroup difference became less significant in middle and higher grades.

**Table 2:** Relationship between various rivalry-state visual task test results and standardized Chinese language scores.

	Standardized Chinese language score			
	Spearman's correlation	Fail Group	Pass Group	Independent T-test
	ρ [*P*]	n [Mean ± SD]	n [Mean ± SD]	t [*P*]
Rivalry-cursor visual acuity test	− 0.023 [0.462]	485 [− 0.02 ± 1.50]	559 [− 0.02 ± 1.32]	− 0.05 [0.96]
Rivalry-simultaneous vision test	0.017 [0.586]	427 [− 0.08 ± 1.59]	617 [0.02 ± 1.27]	− 1.042 [0.298]
Rivalry-fusion vision test	0.011 [0.713]	412 [− 0.12 ± 1.93]	632 [0.04 ± 0.92]	− 1.566 [0.118]
Rivalry-stereoscopic vision test	0.077 [0.013*]	474 [− 0.16 ± 1.85]	570 [0.09 ± 0.87]	− 2.706 [0.007**]

*P < 0.05, **P < 0.01.

**Table 3:** Comparison of standardized chinese language scores between pass and fail groups in rivalry-stereoscopic vision tests across different grades.

	Rivalry-stereoscopic vision test		
	Fail group	Pass group	Independent T-test
	n [Mean ± SD]	n [Mean ± SD]	t [*p*]
Grade 1	102 [− 0.49 ± 3.42]	109 [0.22 ± 0.58]	− 2.081 [0.04*]
Grade 2	99 [− 0.12 ± 1.22]	115 [0.13 ± 0.67]	− 1.84 [0.068]
Grade 3	85 [− 0.04 ± 0.90]	105 [0.10 ± 0.90]	− 1.079 [0.282]
Grade 4	63 [− 0.11 ± 1.01]	85 [0.02 ± 1.24]	− 0.705 [0.482]
Grade 5	84 [− 0.04 ± 1.06]	110 [-0.01 ± 1.00]	− 0.178 [0.859]
Grade 6	41 [0.02 ± 1.19]	46 [0.04 ± 0.64]	− 1.000[0.921]

*p < 0.05.

Table 4 presents the correlation between various rivalry-state visual task tests and standardized mathematics scores, as well as the comparison between the pass and fail groups. We did not find any correlation between the results of these rivalry-state visual task tests and standardized mathematics scores.

**Table 4:** Comparison of standardized mathematics scores between pass and fail groups in rivalry-stereoscopic vision tests across different grades.

	Standardized mathematics score			
	Spearman's correlation	Fail group	Pass group	Independent T-test
	ρ [*P*]	n [Mean ± SD]	n [Mean ± SD]	t [*P*]
Rivalry-cursor visual acuity test	− 0.008 [0.797]	485 [− 0.02 ± 1.05]	559 [− 0.05 ± 1.73]	0.336 [0.737]
Rivalry-simultaneous vision test	0.009 [0.765]	427 [− 0.01 ± 1.02]	617 [− 0.05 ± 1.70]	0.467 [0.641]
Rivalry-fusion vision test	0.024 [0.435]	412 [− 0.13 ± 2.00]	632 [0.03 ± 0.96]	− 1.508 [0.132]
Rivalry-stereoscopic vision test	0.041 [0.183]	474 [− 0.10 ± 1.87]	570 [0.02 ± 1.00]	− 1.447 [0.148]

## Discussion

Primary school students frequently face complex visual environments in reading, writing, and other learning activities, yet traditional physical examinations' vision function tests are limited to single-task assessments, which cannot accurately evaluate students' actual visual performance in real environments. This study aims to develop a simple and realistic rivalry-state visual task test scheme that can rapidly and extensively assess students' performance when encountering complex visual challenges and predict the risk of vision problems. In the four rivalry-state visual task tests conducted in this study, the overall pass rate of students was between 50 and 60%, indicating that nearly half of the students demonstrated poor visual processing ability in challenges similar to complex environments. Although more basic abilities, such as Rivalry-cursor acuity and Rivalry-simultaneous vision, showed a slight increasing trend with grade advancement (Spearman's rank correlation coefficients of 0.114 and 0.108, respectively), this trend does not fully explain an improvement in students' ability to handle visual challenges with age. For more advanced visual functions, such as Rivalry-fusion and Rivalry-stereoscopic vision, the pass rates fluctuated randomly across all grades, with no observed improvement with age.It is important to note that failing the test does not directly imply poor visual function in students. Our study's objective in assessing students' ability to handle visual challenges in complex environments focuses on the risk of developing visual problems under visual stress. In recent years, primary school students have been engaging in near-distance visual tasks for increasingly longer periods, and changes in hemodynamics during visual fatigue can affect normal visual perception^29^. Our screening results serve as a reminder for these students to pay attention to their visual health and avoid excessive eye strain.

In our research findings, although the test results of most rivalry-state visual tasks did not impact academic performance, the Rivalry-stereoscopic vision test was an exception. We discovered that Rivalry-stereoscopic vision significantly affected the Chinese language scores of lower-grade students, particularly new first graders. Students who failed this test scored significantly lower in Chinese than those who passed. As students progress through grades, this impact might gradually be compensated by other external factors (Fig. 2). The influence of Rivalry-stereoscopic vision on lower-grade students' Chinese scores suggests that this type of visual processing ability might be involved in the reading and comprehension of texts. One possible explanation is that children with stronger stereoscopic vision abilities may also have better control over eye movements during reading^30^. Another explanation could be that superior advanced visual processing skills might facilitate the formation of reading-related cross-regional neural networks, especially those responsible for language processing, visual information decoding, and cognitive function integration^31,32^.

This study has certain limitations. Firstly, we only added binocular rivalry-state tasks to classic visual indicators to simulate complex environments, which were limited to simulating contour fusion challenges. Although contour fusion challenges are sufficient to activate advanced visual processing mechanisms in the brain^23–26^, we plan to incorporate other types of visual challenge elements, such as blurriness, noise, distortion, etc., into the design of the visual screening model, to more realistically simulate complex visual environments under quantified experimental conditions. Secondly, our study selected only a limited set of four classic indicators. Future research should be guided by the characteristics of Rivalry-stereoscopic vision tasks and design more in-depth tests to help doctors and educators more accurately identify potential visual problems affecting learning performance. Early detection and appropriate measures can reduce the risk of visual problems affecting academic performance. Lastly, although our designed screening project can support various 3D display devices, we chose the option of wearing red-blue glasses to view a regular monitor, considering the widespread applicability of the screening project, as many schools and hospitals do not have 3D displays.

## Conclusion

This study, by developing a rivalry-state visual task test scheme that simulates real visual environments, provides a new perspective for assessing the performance of primary school students when facing complex visual challenges. Our research finds that nearly 50% of primary school students perform poorly in rivalry-state visual tasks, suggesting their weak adaptability to complex visual environments, and this adaptability does not generally improve with grade advancement. Additionally, our study reveals that deficits in Rivalry-stereoscopic vision might adversely affect the reading and cognitive subject scores (such as Chinese) of children who have just started school. In the current context of increasing near-distance visual tasks and high prevalence of visual disorders, our research highlights the importance of paying attention to primary school students' visual adaptability in complex visual environments.

## Data Availability

The data underlying this article were provided by Yu-Jing Tian with permission. Data will be shared upon request to the corresponding author with permission from Yu-Jing Tian.
